# Grape seed proanthocyanidin extract inhibits ferroptosis by activating Nrf2/HO-1 and protects against diabetic kidney disease

**DOI:** 10.1371/journal.pone.0336472

**Published:** 2025-12-11

**Authors:** Tingting Li, Hao Wang, Baolei Chang, Heng Lu, Ruirui Yang, Haowei Zhang, Haiyan Li, Yusong Ding

**Affiliations:** 1 College of Public Health, Xinjiang Medical University, Urumqi, China; 2 Department of Public Health, Shihezi University School of Medicine, Shihezi, China,; 3 School of Exercise Science, Physical and Health Education, University of Victoria, Victoria, Canada; Helwan University, EGYPT

## Abstract

**Background:**

The main pathological characteristic of diabetic kidney disease (DKD) is renal dysfunction caused by tubular injury. Ferroptosis is a recently discovered form of cell death closely linked to renal tubular injury in DKD. Nuclear factor erythroid-2 related factor 2/heme oxygenase 1(Nrf2/HO-1) is crucial in controlling ferroptosis. Grape seed proanthocyanidin extract (GSPE) mitigates renal dysfunction in DKD by activating Nrf2/HO-1 pathway. However, the role of GSPE in protecting against DKD through ferroptosis modulation has been insufficiently explored.

**Methods:**

Streptozotocin (STZ)-induced diabetic rat models and HK2 cells cultured with high glucose were used as experimental objects in this study. HE and PAS staining was used to observe the morphological changes of rat kidney. Fe^2+^ and reactive oxygen species (ROS) levels as well as apoptosis were determined by fluorescent staining. Transferrin receptor protein 1(TfR1), acylcoa synthetase long chain family member 4(ACSL4), glutathione peroxidase 4 (GPX4), Nrf2 and HO-1 proteins were detected by western blot. Subsequently, Nrf2 was knocked down and oxidative stress and ferroptosis were observed in HK2 cells.

**Results:**

In vivo and vitro result showed that GSPE treatment significantly lessened renal impairment, oxidative stress, and ferroptosis in DKD, while the application of Ferrostatin-1 (Fer-1) notably amplified the anti-ferroptotic effect of GSPE. Moreover, the anti-ferroptotic effect of GSPE was markedly diminished after Nrf2 expression was downregulated in HK2 cells.

**Conclusion:**

GSPE treatment reduced ferroptosis in DKD by modulating the Nrf2/HO-1. In conclusion, our results underscore the significance of ferroptosis in DKD and present new perspectives on the protective effects of GSPE in this condition.

## Introduction

Globally, around 30% to 40% of the 400 million individuals with diabetes mellitus suffer from diabetic kidney disease (DKD) [1]. It is a major cause of end-stage renal disease [2]. Despite the absence of effective pharmacological treatments, the incidence and prevalence of DKD continue to rise. Thus, there is an urgent need to explore more effective strategies to prevent or delay the progression of DKD.

The pathogenesis of DKD is intricate, with hyperglycemia identified as the primary initiating factor [3]. Numerous studies have demonstrated that kidney injury due to elevated glucose levels is associated with an excess of reactive oxygen species (ROS) [4]. These ROS induce lipid peroxidation and mitochondrial dysfunction. Oxidative stress is critical in the advancement of DKD [5]. Under oxidative stress, nuclear factor erythroid-2-related factor (Nrf2) translocates to the nucleus, activates antioxidant response elements, drives its downstream target heme oxygenase 1 (HO-1) and antioxidant enzyme NAD(P)H dehydrogenase quinone 1 (NQO1), attenuates ROS, and maintains cellular redox homeostasis [6]. Therefore, reducing oxidative stress emerges as a viable therapeutic approach for DKD.

Ferroptosis represents a regulated form of cell death caused by the accumulation of iron-dependent lipid reactive oxygen species [7]. This process can be mitigated by blocking lipid peroxidation or reducing iron levels through pharmacological or genetic interventions. Research indicates that cellular iron overload plays a key role in DKD. Iron overload can exacerbate DKD by increasing ROS production and lipid peroxidation accumulation through the Fenton reaction [8]. When ROS levels rise, Nrf2 is activated, further affecting the expression of the downstream target HO-1. Research has confirmed that the Nrf2/HO-1 signalling pathway inhibits ferroptosis [9] and protects renal tubule function in diabetic models [9]. Thus, enhancing Nrf2 expression to reduce ferroptosis is crucial for alleviating DKD progression, and targeting ferroptosis presents a promising strategy for managing DKD.

GSPE is a polyphenolic compound derived from grape seeds. Recent studies have highlighted its pharmacological benefits, particularly its free radical scavenging, antioxidant, and anti-inflammatory effects [10,11]. Previous research indicates that GSPE mitigates renal damage in diabetic rats by activating the Nrf2/HO-1 signaling pathway [12]. However, the effects of GSPE on DKD remain unclear, particularly regarding its potential role in the Nrf2/HO-1 pathway and the underlying mechanisms of high glucose-induced ferroptosis in renal cells. Therefore, this study seeks to investigate whether GSPE can delay DKD progression by inhibiting diabetes-induced ferroptosis via the Nrf2/HO-1 pathway. Our results promise to enhance understanding of DKD prevention through natural phytochemicals.

## Materials and methods

### Source and compositional analysis of the GSPE

GSPE was purchased from Solarbio (SP8520, Beijing) with a purity of 95%. According to the liquid chromatography analysis results provided by the company, GSPE monomer mainly includes four components, including: gallic acid 0.07%, catechin 1.79%, epicatechin 0.99%, epicatechin gallate 0.11%, and oligo proanthocyanin 67.1%.

### Animal experiment

Forty-five healthy male Sprague-Dawley (SD) rats (SPF grade, 8 weeks old, weighing 200 ± 10 g) were purchased from Xinjiang Medical University. The study protocol was approved by the Ethics Committee of First Affiliated Hospital of Shihezi University Medical College (A2018-098-01). The rats were kept under standard laboratory conditions for one week of domestication. At 9 weeks of age, 10 rats (controls) were randomly assigned to a normal diet group, while the remaining 35 rats were fed a high-fat-high-glucose diet. The rats were continuously fed the normal diet and the high-fat-high-glucose diet until the experiment was completed. After 4 weeks, rats on a normal diet received intraperitoneal injections of 1% citrate buffer (Macklin, Shanghai, China; pH 4.50), whereas those on a high-fat-high-glucose diet received intraperitoneal injections of a low dose of Streptozotocin (STZ, Sigma, USA; 40 mg/kg). Two injections were given at 3 day intervals. The rats with fasting blood glucose levels above 16.70 mM on the third day after STZ injection were considered diabetic models. Rats that met the blood glucose criteria were continued to be fed high-fat-high-glucose diet chow for 2 weeks, and 24 hours urine albumin > 30 mg/d was measured as the criterion for successful DKD [13,14]. Due to the fact that DKD rats were left untreated for a long period of time during the experiment, a total of 31 rats were eventually successfully established as DKD models.

The rats were divided into 4 groups, with the first group being the control rats (n = 10). The second group (DKD, n = 11), high-fat-high-glucose-induced DKD rats. In the third group (GSPE+DKD, n = 10), DKD rats were treated with GSPE (Solarbio, Beijing, > 95%). In the fourth group (Fer-1 + DKD, n = 10), DKD rats were treated with Fer-1 (MCE, USA). The control and DKD groups were injected intraperitoneally with deionized water daily, and the GSPE (250 mg/kg) [15–17] group and Fer-1 group (2.50 μmol/kg) [18,19] were injected intraperitoneally with the corresponding measurements daily for 12 weeks. The injection dose and injection time were determined according to the previous study of the group [20]. During the experiment, the rats were free to drink and eat, and their body weight and blood glucose were monitored at regular intervals every week, and their daily activities, water intake, urine intake, and food intake were recorded.

At the end of the study, rats were fasted for 12 hours and urine was collected via metabolic cages. After 18 hours of fasting, mice were anaesthetised by intraperitoneal injection of sodium pentobarbital and rats were executed by taking cervical dislocation. Central venous blood was taken and centrifuged at 3000 rpm/min for 20 min at 4°C and stored in a refrigerator at −80°C for further analysis. Ophthalmic scissors were used to cut open the abdominal cavity to expose the renal tissues, and after the bilateral renal tissues were removed, they were rinsed using pre-cooled saline prepared in advance in a 4°C refrigerator. A portion of the renal tissue samples were fixed in 4% paraformaldehyde embedded in paraffin wax and subsequently stained in sections and imaged on a microscope. The remaining kidney tissue samples were stored in a −80°C refrigerator for subsequent experiments.

### Cell culture

Human renal proximal tubular epithelial (HK2) cells were selected for this experiment and were purchased from the cell bank of the Shanghai Institutes for Life Sciences, Chinese Academy of Sciences. HK2 cells were cultured in low-glucose DMEM medium (Solarbio, Beijing, China) supplemented with 5% fetal bovine serum (Gibco, USA) and 1% antibiotics (Solarbio, Beijing, China), and the cells were maintained in a constant temperature incubator with 5% CO_2_ at 37℃. To construct the DKD cell model, HK2 cells were treated with HG (Kunchuang Biotechnology, Xian, China; 30 mM) for 24 hours. Then, they were treated with different doses of GSPE for 24 hours and divided into low-dose GSPE (L-GSP, 20 mg/L) and high-dose GSPE (H-GSP, 30 mg/L) groups. Meanwhile, the cell model of DKD treated with Fer-1 (1.20 µM) for 24 hours served as the positive control group.

### Cell transfection

Cells were inoculated into 6-well plates and cultured to 70−80% fusion. Cells were transfected with SiNrf2 or siNC Transfection Liposome 2000 (Invitrogen, USA) according to the siRNA kit instructions (Shanghai Genechem Co., Ltd.). The transfection lasted for 5−6 hours. Then, the cells were divided into control, control+si-nc, control+siNrf2, HG, HG+GSPE, HG+GSPE+si-nc, and HG+GSPE+si-Nrf2 treatments for 24 hours. The Nrf2 gene sequence is: GCAGGACAUGGAUUUGAUUTT (sense5’-3’), AAUCAAAUCCAUGUCCUGCTT (antisense5’-3’).

### CCK8 assay

The CCK8 assay assessed the cytotoxicity of high glucose or GSPE on the HK2 cells. The cells were plated onto the 96-well plates at a density of 3 × 10^3^cells per well and treated with high glucose or different doses of GSPE for 24 hours. Then, 10% CCK8 (biasharp, Anhui, China) was added and incubated at 37°C for 1 hours. The cell viability was calculated according to the manufacturer’s instructions.

### PI staining

The cells were seeded onto the six-well plates at a density of 3 × 10^5^ cells per well and incubated at 37°C for 20–30 minutes with PI staining solution (Solarbio, Beijing, China). Then, the cells were observed with a fluorescence microscope (Leica S6E, Wetzlar, Germany).

### HE and PAS staining

Embedded tissue sections were taken and processed according to the instructions of the HE and PAS staining kits (Solarbio, Beijing, China).

### Determination of GSH, MDA, and SOD Levels

The levels of glutathione (GSH), malondialdehyde (MDA), and Superoxide Dismutase (SOD) were measured using the corresponding kits (Nanjing Jiancheng Bioengineering Institute, China) according to the manufacturer’s instructions.

### ROS activity detection

HK2 cells were inoculated into 6-well plates at a density of 3 × 10^5^ cells per well. The treated cells were incubated with dichlorodihydrofluorescein-diacetate (DCFH-DA) (1 μM) for 30 minutes at 37°C according to the instructions of the ROS kit (Nanjing Jiancheng Bioengineering Institute, China) and then rinsed with PBS. All fluorescent images of the cells were observed using a fluorescence microscope (Leica S6E, Wetzlar, Germany).

### Measurement of iron accumulation

According to the kit instructions, the Lillie Staining Assay kit (Solarbio, Beijing, China) was used to detect divalent iron deposition in renal tissues and cellular divalent iron. The observation was performed with the fluorescence microscope (Leica S6E, Wetzlar, Germany). The Image J software was used to perform the image analysis.

### Western blot analysis

Total proteins from collected cells and kidney tissues were extracted using a radioimmunoprecipitation assay (RIPA) lysis buffer (Solarbio, Beijing, China).Then, the Nucleic Acid Meter (Thermo Fisher Scientific) determines the protein concentration of the sample, and after determining the volume and concentration of the sample, the concentration of the sample is levelled using the levelling formula.Subsequently, an immunoblot assay was performed as previously described [21].The primary antibodies against GPX4 (ab125066, 1: 2000), TfR1 (ab129177, 1: 1000), ACSL4 (ab129177, 1: 20000), HO-1 (ab68477, 1: 1000), NQO1 (ab80588, 1: 1000), Nrf2 (ab137550, 1: 1000) were purchased from Abcam. PTGS2 (A00084-2, 1:1000) purchased from Wuhan Doctor De Co., Ltd. Horseradish peroxidase-conjugated goat anti-mouse/rabbit IgG and mouse anti-β-actin (1: 1000) monoclonal antibodies were acquired from (ZSGB-BIO, Beijing, China).

### Detection of mitochondrial membrane potential

HK2 cells were inoculated into 6-well plates at a density of 3 × 10^5^ cells per well. The cells were stained with 20 μM JC-1 for 45 minutes at 37°C in the dark according to the instructions of the JC-1 kit (Solarbio, Beijing, China). The cells were then washed twice with JC-1 buffer. Finally, cells were observed with a fluorescence microscope (Leica S6E, Wetzlar, Germany).

### Statistical analysis

Data from three independent experiments were expressed as mean±standard deviation (SD). The SPSS 20.0 software was used for data analysis. One-way ANOVA followed by the Bonferroni test or t-test was used to examine the data statistically. A 95% confidence interval was utilized for all statistical tests. *P* < 0.05 was considered statistically significant.

## Results

### 1. GSPE mitigated kidney injury in DKD rats

**Fig 1:**
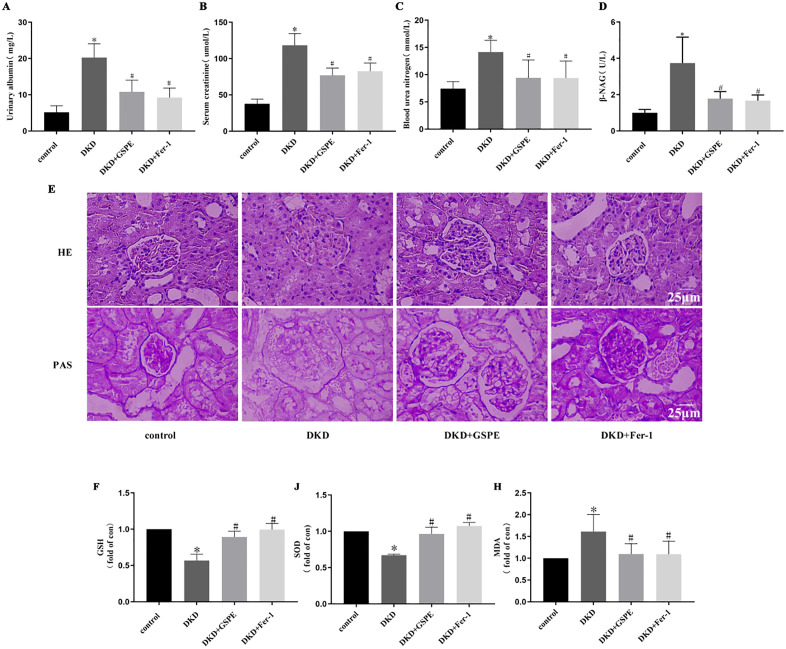
Impacts of GSPE on physicochemical characteristics and renal morphological changes in DKD rats. (A) The levels of UAD content. (B) The levels of Scr content. (C) The levels of BUN content. (D) The levels of β-NAG content. (E) Renal HE staining and PAS staining results (400×). (F) The levels of GSH content. (J) The levels of SOD content. (H) The levels of MDA content. Data are expressed as mean±standard deviation (* *p* < 0.05 vs. control group; # *p* < 0.05 vs. DN group, n = 3).

To evaluate the effect of GSPE on kidney injury in DKD rats, urinary albumin (UAD), serum creatinine (Scr), blood urea nitrogen (BUN) and N-acetyl-β-D-glucosidase (β-NAG) were measured (Fig 1A-D). UAD, Scr, BUN and β-NAG were significantly increased in DKD rats. GSPE and Fer-1 treatment significantly improved the level of urinary albumin, serum creatinine, blood urea nitrogen and β-NAG in DKD rats.The effects of GSPE and Fer-1 on morphological changes in DKD rats were then observed by HE and PAS staining (Fig 1E). HE and PAS staining showed significant renal pathological changes in DKD rats, including glomerular hypertrophy, mesangial matrix dilatation, renal tubule lumen expansion, vacuolar degeneration, and exfoliation of some renal tubular epithelial cells with the disappearance of brush margins. After GSPE and Fer-1 treatment, these kidney damage changes were dramatically improved.

It was found that GSPE plays a key role in antioxidant defense [22]. So we examined the related indexes of antioxidant defense, and the data showed that GSH and SOD levels were significantly decreased, and MDA levels were significantly increased in the kidneys of DKD rats compared with the control group, whereas GSPE Fer-1 significantly inhibited the depletion of GSH and SOD levels and the increase of MDA content (Fig 1F-H), which indicated that GSPE could inhibit the oxidative stress damage in the kidneys of DKD rats (S1 Fig-S5 Fig).

### 2. GSPE prevents ferroptosis and activates the Nrf2/HO-1 pathway in the kidney of DKD rats

**Fig 2:**
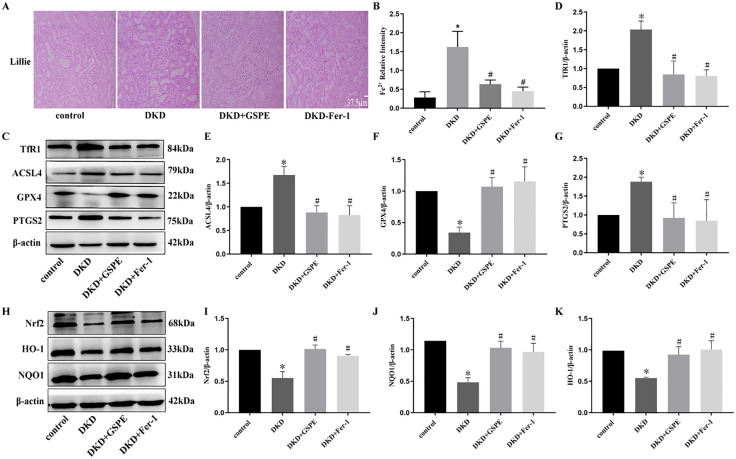
GSPE prevents ferroptosis and activates the Nrf2/HO-1 pathway in the kidney of DN rats. (A). Detection of ferrous iron content in kidney tissue using the Lillie staining method (400×). (B). Quantified ferrous iron content. (C-G). The expression and quantification of ACSL4, TfR1, GPX4, and PTGS2 protein. (H-K). The expression and quantification of Nrf2, HO-1 and NQO1 protein. Values were expressed as mean±SD (* *p* < 0.05 vs. the control group; # *p* < 0.05 vs. the DN group, n = 3).

To verify whether ferroptosis is involved in the function of GSPE, Fer-1, an activator of ferroptosis and GSPE was used to treat DKD rats. We detected iron levels in the kidney of DKD rats, compared to the control group, Lillie staining revealed that iron content was significantly elevated, GSPE and Fer-1 treatment significantly reduced the mean density of iron content (Fig 2A-B). Next, we explored the underlying mechanism by which regulatory proteins regulate iron intake and release. Western blot analysis confirmed the high level of TfR1 in the kidney tissue of DKD rats. GSPE and Fer-1 treatment significantly reduced the level of TfR1 protein. Meanwhile, we evaluated the expression of ACSL4, GPX4, and PTGS2, the classic markers of ferroptosis. Western blot analysis showed that GPX4 levels significantly declined in the DKD group, ACSL4 and PTGS2 levels were significantly increased in the DKD group (Fig 2C-G). In comparison, GSPE and Fer-1 reversed these changes. These results suggested that GSPE and Fer-1 may attenuate ferroptosis in the kidney of DKD rats.

Previous studies have found that Nrf2 pathway can regulate ferroptosis-related proteins [23], and this study found that GSPE can inhibit ferroptosis, but whether GSPE inhibits ferroptosis by activating the Nrf2 pathway is not clear, in order to explore the role of GSPE in the renal tissues of DKDrats, this study examined the expression of the Nrf2 pathway-related proteins in the renal tissues of rats with DKD in each group. Western blotting results showed that the expression of Nrf2, HO-1and NQO1 was reduced in the DKD group compared with the control groupt, the expression of Nrf2, HO-1, and NQO1was elevated after intervention with GSPE and Fer-1 compared to the DKD group (Fig 2H-K). The above findings suggest that GSPE activated the Nrf2 pathway and increased the expression levels of its downstream HO-1, NQO1. Based on the above findings in this study, we speculate that GSPE may inhibit iron death and improve the antioxidant capacity of renal tissues in DKD rats by activating the Nrf2 antioxidant pathway. In order to investigate the mechanism of action of GSPE on renal injury in DKD rats, we further investigated it in cellular experiments.

### 3. GSPE plays a protective role in HG-induced renal tubule injury via inhibiting ferroptosis

**Fig 3:**
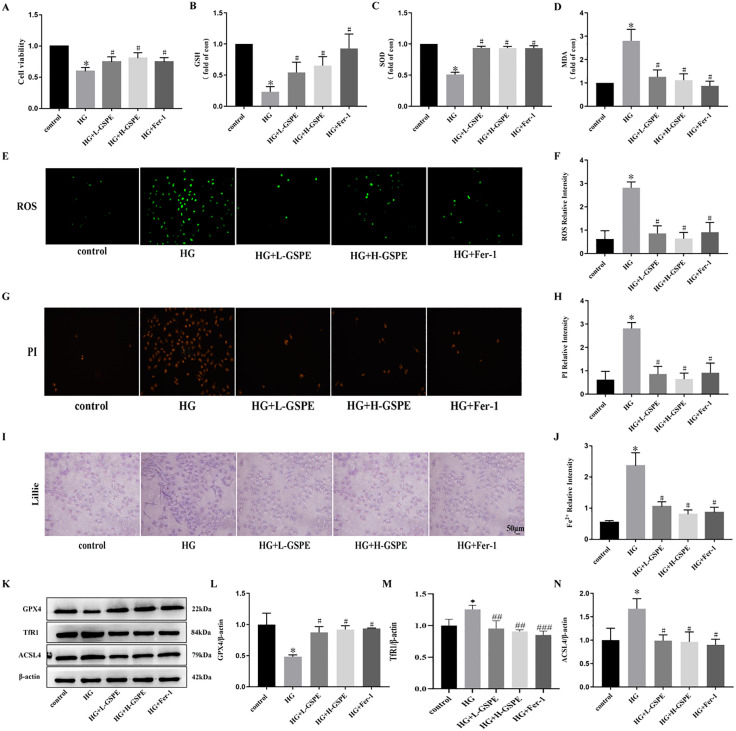
GSPE plays a protective role in HG-induced renal tubule injury via inhibiting ferroptosis. (A). The effect of cell viability. (B). The levels of GSH content. (C). The levels of SOD content. (D). The levels of MDA content. (E). ROS levels detected by DCFH-DA staining (100×). (F). Relative ROS intensity. (G). PI staining was performed to detect the death of HK2. The PI-positive nuclei were stained red (200×). (H). Relative PI intensity. (I). HG induces changes in intracellular Fe^2+^ content in HK2 cells (100×). (J). Relative Fe2+ intensity. (K-N). The expression and quantification of GPX4, TfR1and ACSL4 protein.Values were expressed as mean±SD (* *p* < 0.05 vs. the control group; # *p* < 0.05 vs. the HG group, n = 3).

HK2 cells were cultured with 30 mM glucose to establish an in vitro experimental model to explore the function of GSPE in HG-induced renal tubule injury. Different concentrations of GSPE (20, 30 mg/L) and Fer-1 (1.20 μM) were applied to HG-induced HK-2 cells. Compared to the control group, the HG group’s cell viability significantly decreased (Fig 3A). We also found that the GSH content and total SOD were lower in the HG group than the control group (Fig 3B-C), and MDA and ROS content was significantly elevated in the HG group than in the control group (Fig 3D-F). In comparison, GSPE reversed these changes, these results suggest that GSPE reduces oxidative stress in HG-induced HK-2 cells. Additionally, We examined PI staining, iron content and typical markers of iron prolapse in HG-induced HK2 cells, which showed increased cell death (Fig 3G-H), increased Fe^2+^ content (Fig 3I-J), decreased expression of iron death-associated proteins GPX4 and TfR1, and increased expression of ACSL4 (Fig 3K-N) in the HG group compared to the control group. The response to the above changes after GSPE treatment was in line with the role of Fer-1 consistent. This suggests that GSPE can inhibit HG-induced ferroptosis in HK2 cells, consistent with results the in vivo.

### 4. GSPE activated Nrf2/HO-1 pathway in HG-stimulated renal tubular epithelial cells

**Fig 4:**
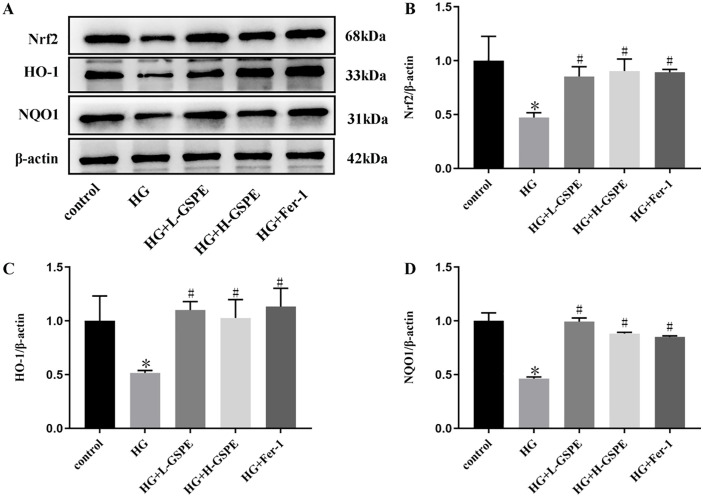
GSPE activated Nrf2/HO-1 pathway in HG-stimulated renal tubular epithelial cells. (A-D). The expression and quantification of Nrf2, HO-1, and NQO1 protein. Values were expressed as mean±SD (* *p* < 0.05 vs. the control group; # *p* < 0.05 vs. the HG group, n = 3).

Given the important role of Nrf2 in ferroptosis, we investigated the effect of GSPE on the Nrf2/HO-1 pathway in HG-induced HK2 cells. The result demonstrated that Nrf2, HO-1, and NQO1 proteins were down-regulated in HG-induced HK2 cells. The expression levels of Nrf2, HO-1, and NQO1 were increased after GSPE and Fer-1 intervention (Fig 4A-D). These are consistent with in vivo results.

### 5. Down-regulation of Nrf2 attenuated the inhibitory effect of GSPE on ferroptosis

**Fig 5:**
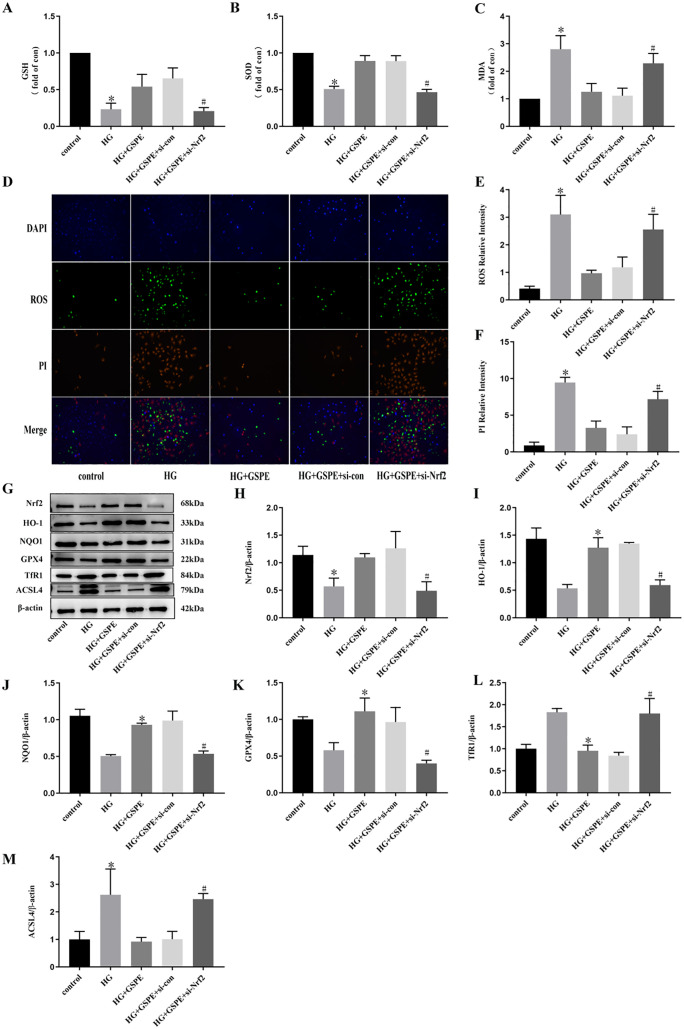
Knockdown of Nrf2 blocked the inhibitory effect of GSPE on ferroptosis in HK2 cells induced by HG. (A). The levels of GSH content. (B). The levels of SOD content. (C). The levels of MDA content. (D). ROS levels detected by DCFH-DA staining (100×) and PI staining was performed to detect the death of HK2, the PI-positive nuclei were stained red (200×). (E). Relative ROS intensity. (F). Relative PI intensity. (G-M). The expression and quantification of Nrf2, HO-1, NOQ1, GPX4, TfR1, and ACSL4. Values were expressed as mean±SD (* *p* < 0.05 vs. the control group; # *p* < 0.05 vs. the HG group, n = 3).

To determine whether the protective effect of GSPE on ferroptosis in HK2 cells stimulated by HG involves the activation of Nrf2 signaling, cells were transfected with specific Nrf2 Si-RNA. Consistent with the results of the previous study, SOD and GSH were significantly decreased (Fig 5A-B), while MDA was significantly decreased (Fig 5C), cell death and ROS were significantly increased (Fig 5D-F) under HG stimulation, GSPE reversed the effects of HG and attenuated oxidative stress, and GSPE also rescued the cellular turnover induced by HG. Down-regulation of Nrf2 blocked the action of GSPE, up-regulated SOD and GSH, and down-regulated MDA, cell death and ROS (Fig 5A-F), and increased the rate of cell turnover. In addition, we examined the changes of ferroptosis-related proteins as well as Nrf2/HO-1 signalling pathway-related proteins, and the results showed that TfR1 and ACSL4 proteins were significantly elevated, while GPX4, Nrf2, and HO-1 proteins were significantly decreased under the stimulation of HG (Fig 5G-M). GSPE reversed the action of HG and attenuated ferroptosis. Down-regulation of Nrf2 blocked the action of GSPE, up-regulating TfR1 and ACSL4 protein levels and down-regulating GPX4, NRF2 and HO-1 protein levels. This is further evidence that GSPE antagonises ferroptosis via the Nrf2/HO-1 pathway.

## Discussion

GSPE has potential clinical values and application prospects as a therapeutic agent for diabetes and its complications, including hypoglycemic, antioxidant, and anti-inflammatory effects [24]. However, multiple modes of action in the impact of GSPE extracts in DKD still need to be illuminated. This study aimed to investigate the protective effect of GSPE against DKD and its potential molecular mechanisms in vivo and in vitro models. Investigating the mechanisms contributing to DKD will be beneficial in controlling this fatal disease. This study showed that GSPE treatment significantly improved urinary albumin, serum creatinine, blood urea nitrogen, and β-NAG levels. These results suggest the protective effect of GSPE on DKD.

Ferroptosis is an iron-dependent form of regulated cell death driven by an overload of lipid peroxides on cellular membranes [25]. It occurs when ferroptosis-promoting cellular activities significantly override the antioxidant-buffering capabilities provided by ferroptosis defense systems [26]. ACSL4 catalyzes the biosynthesis of arachidonic coenzyme A that contributes to the execution of ferroptosis by triggering phospholipid peroxidation [27]. GPx4 is a structural protein and antioxidant enzyme that effectively inhibits lipid oxidation and is considered a key regulator of ferroptosis [28]. TfR1, as one of the key members of the iron ion entry channel into the cell, plays a critical role in the regulation of cellular iron metabolism and the maintenance of iron homeostasis, and is considered to be a marker protein for the occurrence of ferroptosis [29]. This study showed that GPX4 levels significantly declined in the DKD group and ACSL4 and TfR1 levels increased significantly in the DKD group.

Ferroptosis is regulated by iron overload, lipid peroxidation, and glutathione depletion. Maintaining iron and redox homeostasis is essential for the normal function of renal cells [30]. Iron overload and hyperglycemia-mediated ROS accumulation in renal tubule cells are vital components of the pathogenesis of DKD [31]. The traditional pathogenesis of DKD is involved in oxidative stress [32]. Our results showed that iron overload led to an increase in ROS and lipid peroxidation product MDA, in addition to a decrease in antioxidants GSH and SOD, in high glucose-induced HK2 cells in renal tubules of DKD rats. These results suggest that ferroptosis may play a pathological role in the development of DKD through oxidative stress.

Ferroptosis can be suppressed by blocking lipid peroxidation directly or through depleting iron via pharmacological or genetic means [33]. Nrf2 is a transcription factor that plays an essential role in cellular antioxidant activity and regulates the transcription of components of the GSH antioxidant systems and heme metabolism enzymes [34]. As many of its downstream genes are associated with ferroptosis, Nrf2 is considered a critical regulator of ferroptosis [23]. Previous studies have shown that activation of the Nrf2/HO-1 signaling pathway protects renal oxidative damage in diabetic models [17]. Since GSPE acts as a regulator of the Keap1/Nrf2/ARE signaling pathway [12], we hypothesized that the Nrf2/HO-1 signaling pathway could serve as a potential therapeutic target for GSPE to delay the progression of DKD. Concordantly, this study found that chronic hyperglycemia inhibited Nrf2 and HO-1 protein levels in an in vitro and in vivo DKD model. On the other hand, GSPE treatment induced the activation of Nrf2 and increased its translocation to the nuclear level, thus promoting the HO-1 protein expression. Our study found that Nrf2, HO-1and NQO1 protein were down-regulated in renal tubules of DKD rats and HK2 cells that were exposed to high glucose; GSPE activated Nrf2/HO-1 pathway in the kidney of DKD rats and HG-stimulated renal tubular epithelial cell; and down-regulation of Nrf2 attenuated the inhibitory effect of GSPE on ferroptosis. These results provide insight into DKD and show that targeting Nrf2 to regulate lipid peroxidation and ferroptosis is a feasible disease intervention strategy.

However, while this study validated that GSPE can activate the Nrf2/HO-1 signalling pathway, the mechanism of action of Nrf2 remains unclear. In our subsequent research, we will further investigate whether Nrf2 undergoes nuclear translocation, clarify how GSPE activates the Nrf2/HO-1 signalling pathway, and explore its potential targets to closely integrate it with clinical treatment of DKD. Additionally, due to rat-specific physiological differences and the lack of complex in vivo microenvironments in cell cultures, translating animal study results and cell culture models into human clinical applications still faces numerous challenges. In our subsequent studies, we will further consider using models that more closely resemble human physiology, such as kidney organoids derived from diabetic kidney disease patients and non-human primate animal models, to validate the efficacy of GSPE.

## Conclusions

In this study, we demonstrated in ex vivo and in vivo experiments that iron death was involved in renal tissue and HK2 cell injury in DKD rats, accelerating the development of DKD. However, GSPE increased antioxidant capacity, improved renal function, reduced iron overload, and inhibited iron death and inflammatory responses, thereby ameliorating DKD renal tissue injury and HG-induced HK2 cell injury, and the specific mechanism may be that GSPE inhibited oxidative stress and iron death through activating the Nrf2/HO-1 signaling pathway and delayed the development of DKD. These results indicate that GSPE has the potential to become a safe and reliable alternative in the clinical treatment of DKD, bringing new hope to patients. Meanwhile, GSPE is expected to become a key ingredient of new anti-diabetic drugs due to its natural, efficient, and low-cost advantages. GSPE is expected to be a key ingredient of new anti-diabetic drugs, which will help the pharmaceutical field to make steady progress in the relevant direction.

## Supporting information

S1 raw images Original images for all blots and gels. (PDF)

S1 Fig Original data of renal function index and renal oxidative injury. (PDF)

S2 Fig Original image of Lillie staining. (PDF)

S3 Fig OriginaldataofHK2cellsurvivalrate,oxidativestress,PIstainingand Lilliestaining. (PDF)

S5 Fig Originaldataofoxidativestress,ROSstainingandPIstaininginHK2 cells. (PDF)
