# Supplementary figures and images for: Grape seed proanthocyanidin extract inhibits ferroptosis by activating Nrf2/HO-1 and protects against diabetic kidney disease
Source: PLoS One. 2025 Dec 11;20(12):e0336472. doi: 10.1371/journal.pone.0336472 (PMC12697995; doi:10.1371/journal.pone.0336472)

Original images for all blots and gels

Figure 2

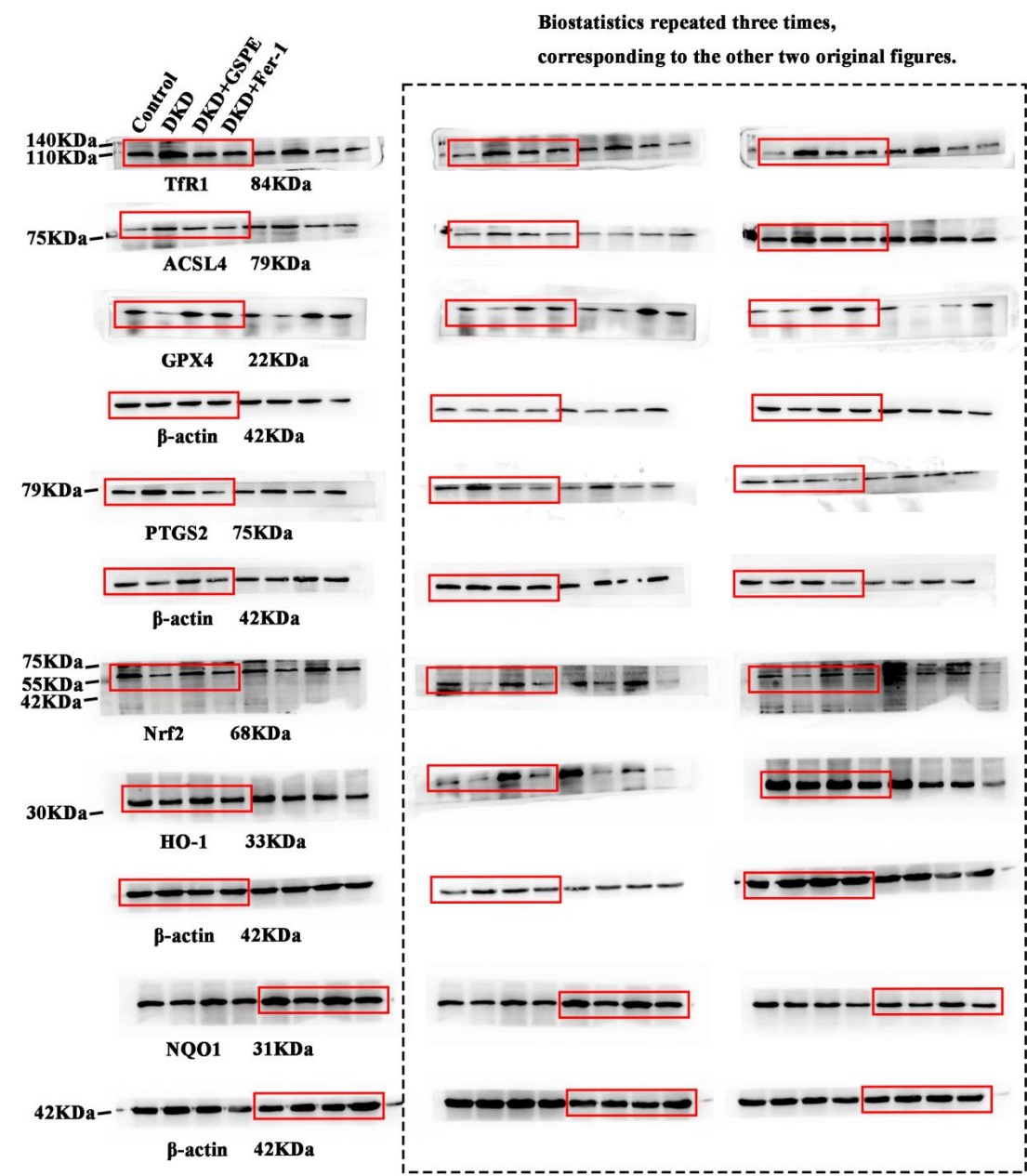

Figure 3 and Figure 4

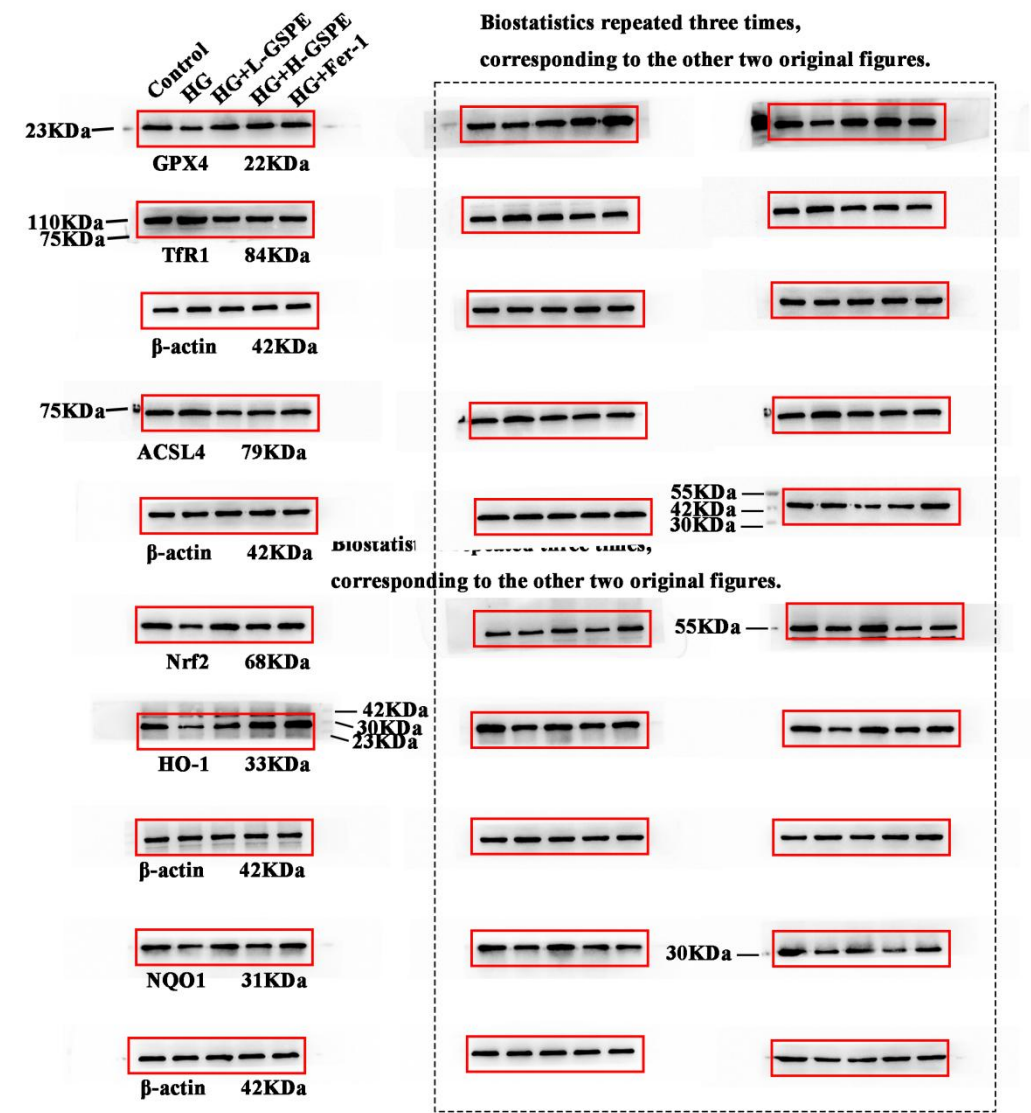

Figure 5

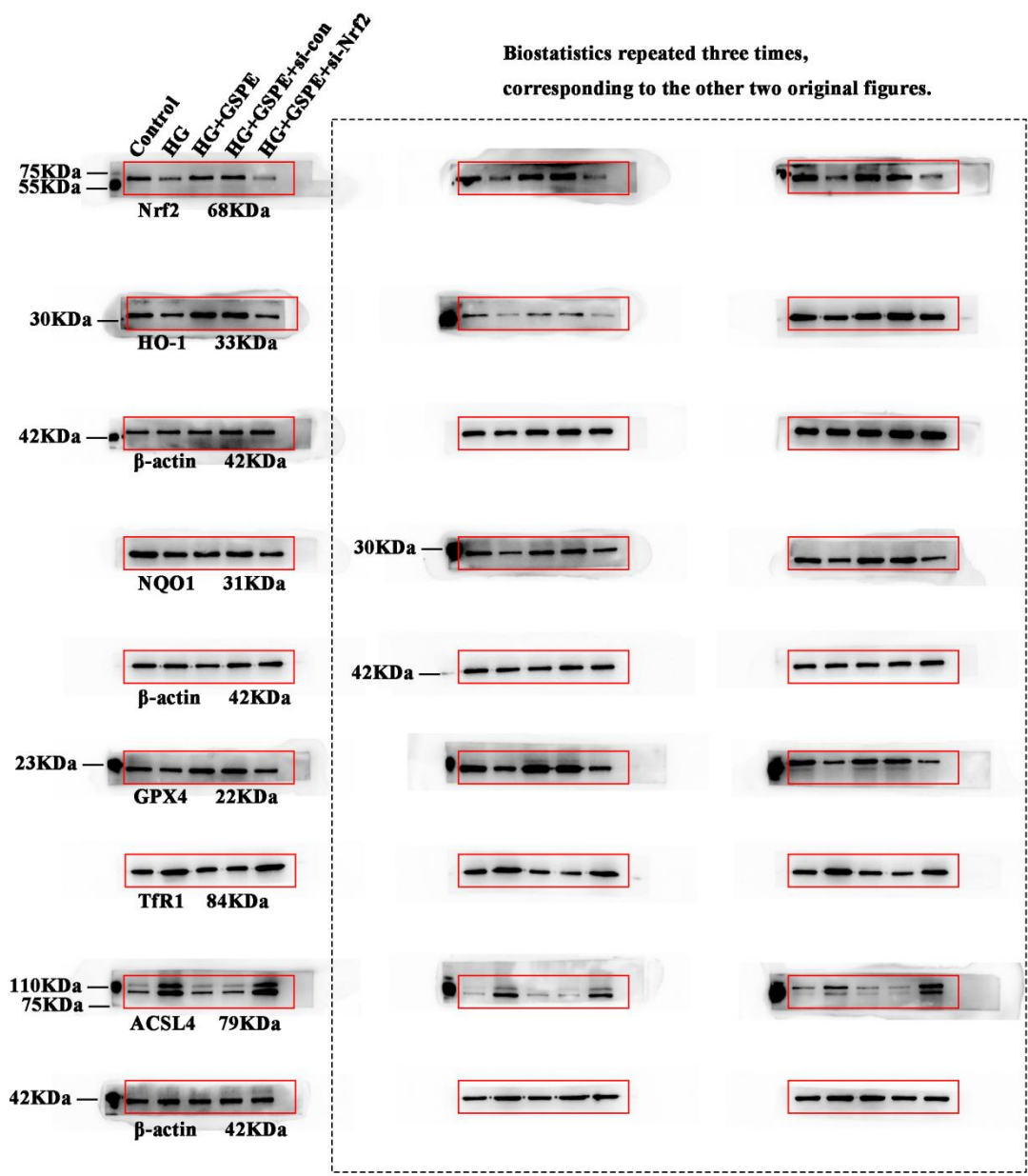

Supplement: S1 raw images — (PDF) [file pone.0336472.s001.pdf]

## Original image of Lillie staining

Figure 2

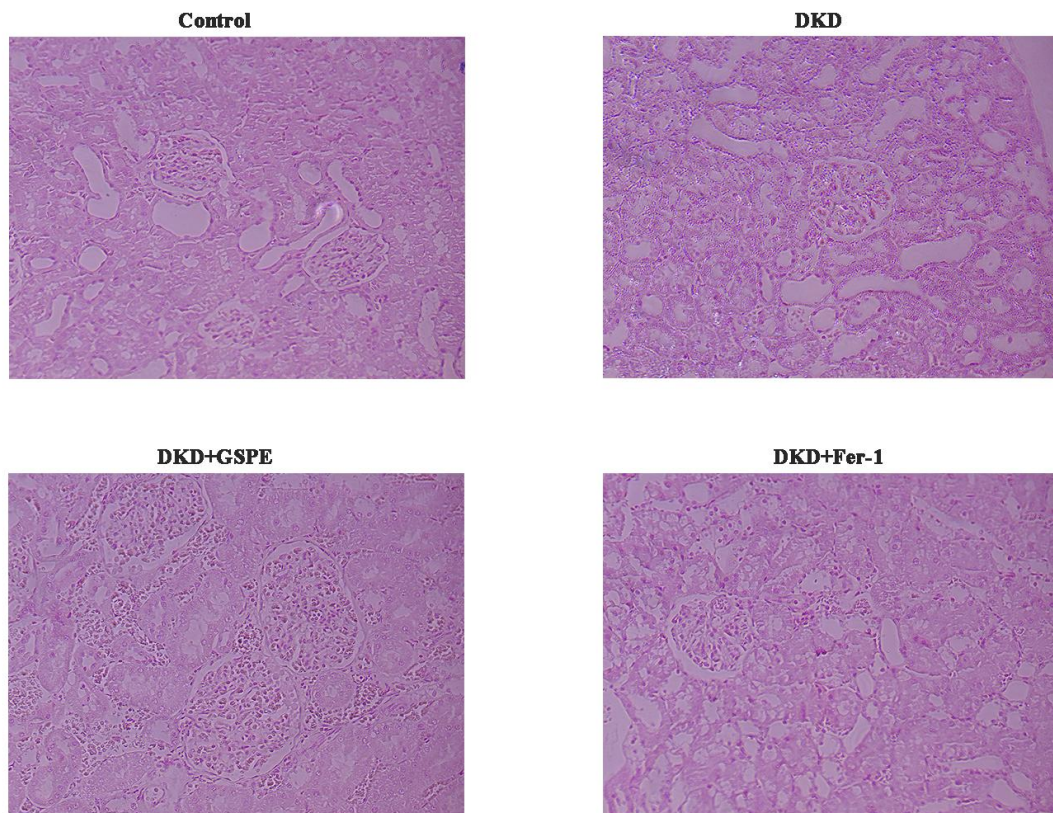

Supplement: S2 Fig — (PDF) [file pone.0336472.s003.pdf]
