# Supplementary material for: Grape seed proanthocyanidin extract inhibits ferroptosis by activating Nrf2/HO-1 and protects against diabetic kidney disease
Source: PLoS One. 2025 Dec 11;20(12):e0336472. doi: 10.1371/journal.pone.0336472 (PMC12697995; doi:10.1371/journal.pone.0336472)
Supplement: S1 Fig — (PDF) [file pone.0336472.s002.pdf]

Original data of renal function index and renal oxidative injury

Figure 1

Urinary albumin			
control	DKD	DKD+GSPE	DKD+Fer-1
4.32	16.49	13.45	8.95
5.25	16.92	8.65	7.26
6.91	20.31	10.26	8.95
7.15	25.64	9.26	9.61
3.21	18.68	11.65	10.29
4.62	18.92	15.64	14.92
2.91	15.93	8.65	11.35
8.34	22.35	9.23	8.26
5.26	27.35	5.92	7.66
3.64	19.99	15.49	5.24

Serum creatinine			
control	DKD	DKD+GSPE	DKD+Fer-1
30.77	110.34	89.43	73.41
43.22	90.63	63.67	69.85
49.03	103.34	90.46	93.21
34.36	140.57	69.15	89.7
26.49	118.49	83.43	100.32
37.55	127.47	62.58	70.56
37.68	130.67	74.33	69.88
41.69	103.51	81.21	84.37
36.87	136.52	73.68	85.44
40.77	122.33	82.32	90.64

Blood urea nitrogen			
control	DKD	DKD+GSPE	DKD+Fer-1
7.62	12.36	8.92	13.52
8.26	16.52	8.97	10.94
9.15	14.38	9.16	8.92
6.35	15.21	10.92	7.64
4.92	11.65	11.84	4.36
6.51	14.35	9.91	9.27
8.16	13.28	4.92	15.18
8.22	14.36	15.36	8.27
6.93	18.19	10.35	7.34
8.49	11.28	3.92	8.49

N-acetyl-β-D-glucosidase			
control	DKD	DKD+GSPE	DKD+Fer-1
0.96	3.35	2.02	1.68
1.10	4.28	2.74	2.13
0.75	3.92	1.48	1.80
1.15	5.16	1.61	1.37
1.31	3.52	1.80	1.42
1.00	3.69	1.90	1.59
0.77	4.30	1.67	2.22
0.80	4.51	1.49	1.71
1.19	4.69	1.40	1.50
0.97	0.00	1.70	1.30

HE stain

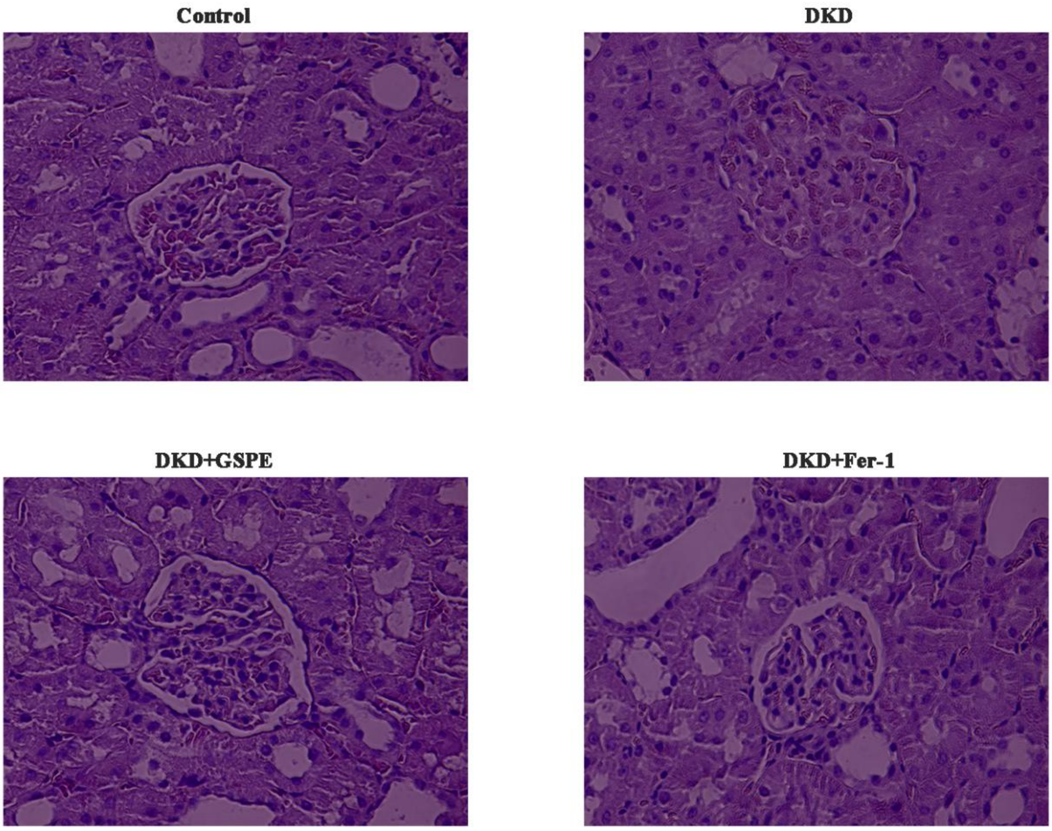

PAS stain

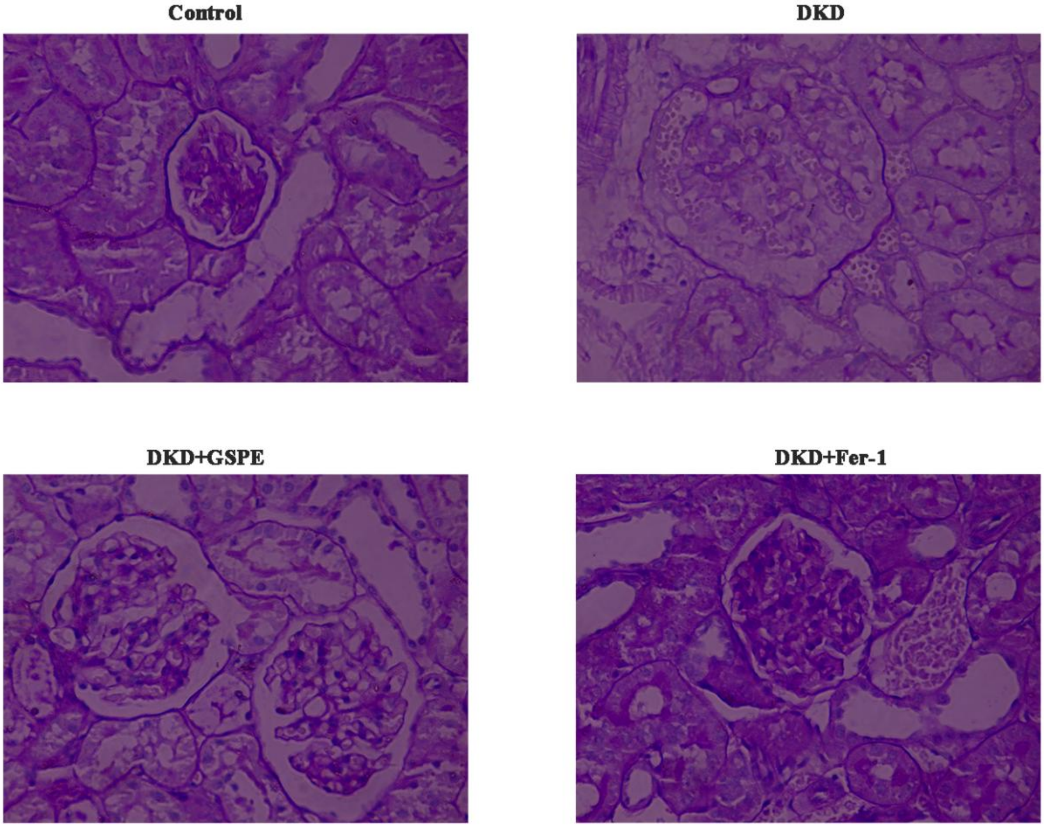

Glutathione			
control	DKD	DKD+GSPE	DKD+Fer-1
114.37	53.18	86.80	99.72
94.92	62.32	91.91	95.94
98.81	63.15	91.06	104.25
97.25	49.86	89.36	103.49
100.37	60.66	91.06	101.98
102.70	61.49	94.46	110.29
109.70	45.70	87.65	105.00
107.37	62.32	91.06	91.41
92.59	60.66	92.76	97.45
102.01	57.71	90.68	101.06

Superoxide Dismutase			
control	DKD	DKD+GSPE	DKD+Fer-1
93.35	61.59	81.32	100.42
96.94	66.89	104.84	109.61
102.24	67.96	95.27	103.69
92.57	61.36	80.94	99.88
96.42	66.36	104.56	108.52
101.25	67.27	94.79	103.20
91.95	60.90	80.60	99.52
96.10	66.20	104.12	107.73
100.83	67.08	94.41	102.54
96.85	65.07	93.43	103.90

Malondialdehyd			
control	DKD	DKD+GSPE	DKD+Fer-1
1.54	2.29	2.01	2.29
1.74	2.96	2.41	1.98
2.83	2.81	2.34	2.19
1.54	3.29	1.61	2.48
1.83	2.96	2.31	2.08
2.24	3.31	1.64	1.79
1.84	3.29	2.44	1.78
1.84	3.96	1.74	2.08
2.24	2.64	2.34	1.78
1.96	3.06	2.10	2.05
