# Supplementary material for: Grape seed proanthocyanidin extract inhibits ferroptosis by activating Nrf2/HO-1 and protects against diabetic kidney disease
Source: PLoS One. 2025 Dec 11;20(12):e0336472. doi: 10.1371/journal.pone.0336472 (PMC12697995; doi:10.1371/journal.pone.0336472)
Supplement: S3 Fig — (PDF) [file pone.0336472.s004.pdf]

Original data of HK2 cell survival rate, oxidative stress, PI staining and Lillie staining

Figure 3

CCK8					
control	HG	HG+L-GSPE	HG+H-GSPE	HG+Fer-1	blank
1.10	0.79	0.79	0.96	0.95	0.18
1.10	0.69	0.96	0.87	0.82	0.17
1.14	0.71	0.85	0.89	0.86	0.18
1.08	0.79	0.80	0.97	0.93	0.18
1.08	0.68	0.93	0.86	0.84	0.17
1.17	0.70	0.85	0.90	0.84	0.18
1.07	0.78	0.80	1.06	0.92	0.18
1.08	0.69	0.96	0.85	0.84	0.17
1.09	0.72	0.85	0.93	0.81	0.18

GSH				
control	HG	HG+L-GSPE	HG+H-GSPE	HG+Fer-1
5.34	0.91	3.72	4.19	6.47
6.59	0.98	2.89	3.63	4.76
5.93	1.32	2.26	3.33	5.37
5.32	1.84	4.01	4.46	6.45
6.36	0.99	2.92	3.68	4.22
5.60	1.60	2.53	3.06	5.10
5.29	1.84	4.30	4.74	6.43
6.36	0.98	3.18	3.58	4.26
5.84	1.60	2.23	3.29	5.02

SOD				
control	HG	HG+L-GSPE	HG+H-GSPE	HG+Fer-1
13.28	6.21	12.33	12.35	9.67
13.03	7.24	12.91	12.44	11.61
13.48	6.86	12.89	12.87	12.89
13.27	6.18	12.21	11.92	12.21
13.03	7.19	12.45	12.68	12.45
13.52	6.84	12.46	12.54	12.46
13.29	6.19	11.92	12.63	11.92
13.03	7.21	12.14	12.03	10.83
13.49	6.85	12.50	12.17	12.50

MDA				
control	HG	HG+L-GSPE	HG+GSPE	HG+Fer-1
3.37	12.28	11.45	4.58	2.76
3.84	8.35	8.87	2.92	2.68
3.98	7.84	8.63	4.24	3.96
3.37	12.17	11.33	4.44	2.76
3.88	8.64	9.27	2.59	2.83
3.61	8.94	8.93	3.75	3.95
3.74	11.68	12.08	4.95	3.13
3.87	9.81	10.52	4.00	2.38
2.88	11.49	9.14	4.18	3.57

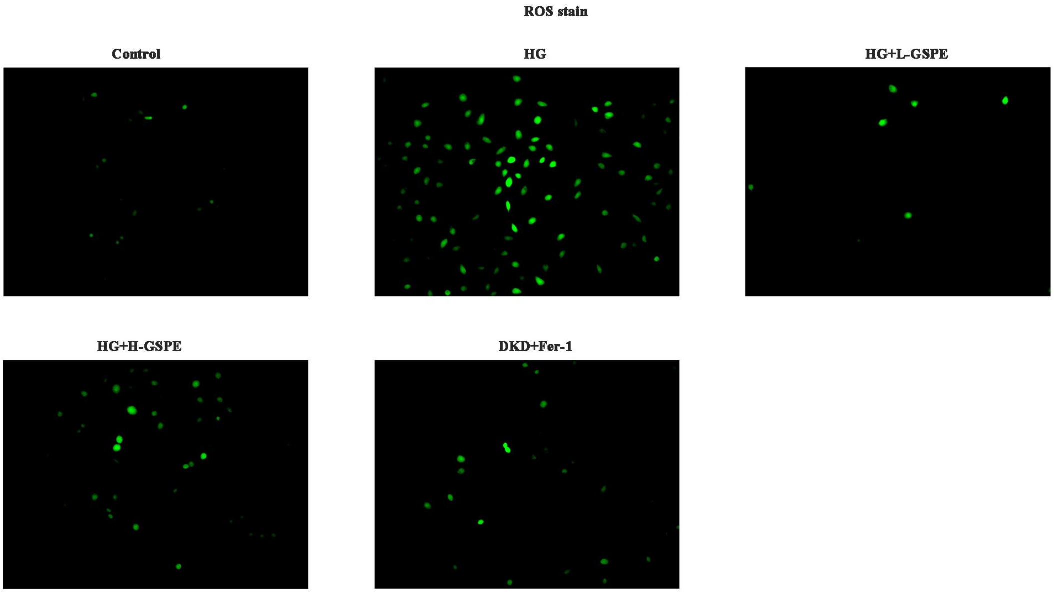

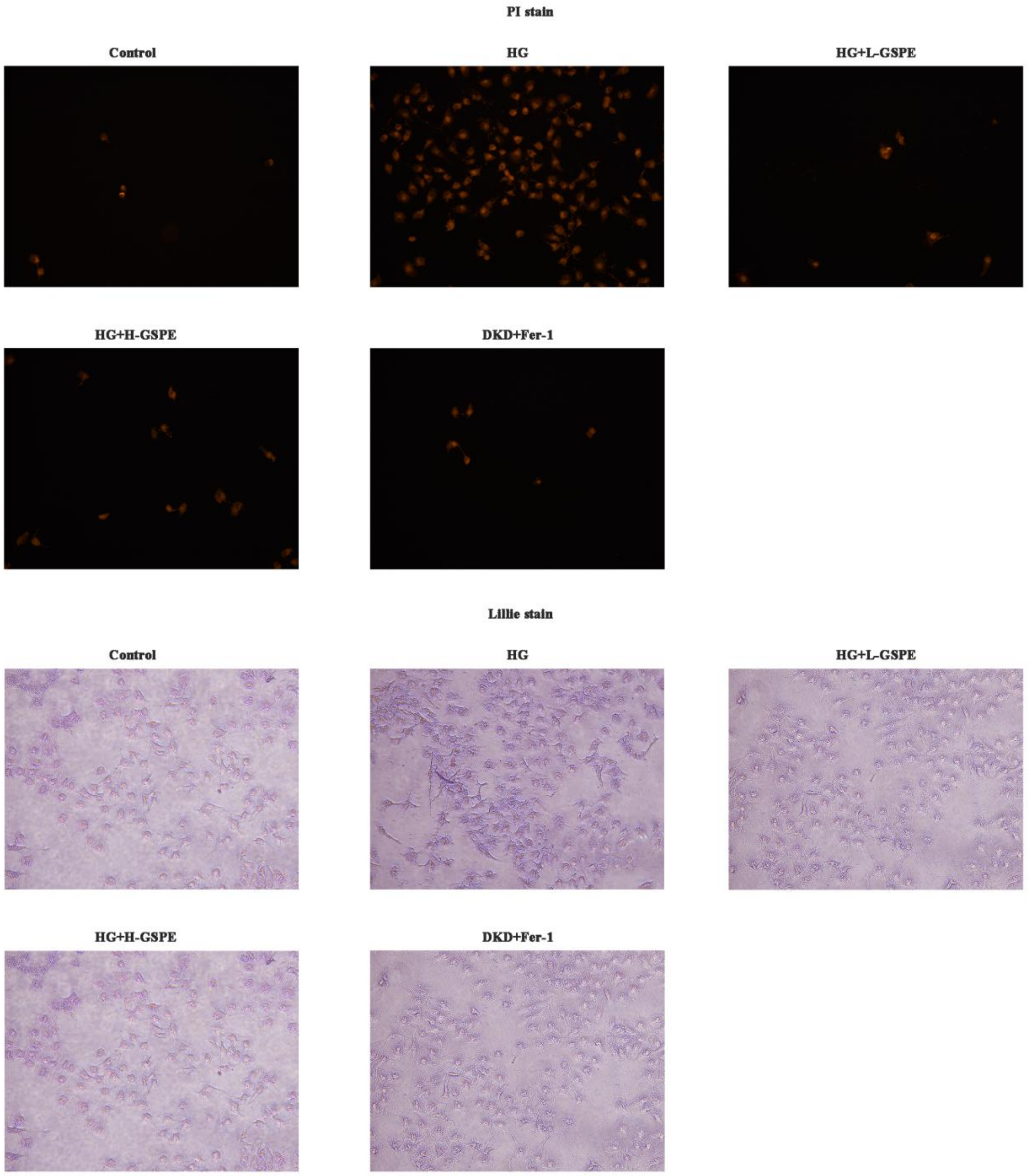

PI stain
Control
HG
HG+L-GSPE
HG+H-GSPE
DKD+Fer-1
Lillie stain
Control
HG
HG+L-GSPE
HG+H-GSPE
DKD+Fer-1
