# Supplementary material for: Grape seed proanthocyanidin extract inhibits ferroptosis by activating Nrf2/HO-1 and protects against diabetic kidney disease
Source: PLoS One. 2025 Dec 11;20(12):e0336472. doi: 10.1371/journal.pone.0336472 (PMC12697995; doi:10.1371/journal.pone.0336472)
Supplement: S5 Fig — (PDF) [file pone.0336472.s005.pdf]

Original data of oxidative stress, ROS staining and PI staining in HK2 cells

Figure 5

GSH				
control	HG	HG+GSPE	HG+GSPE+si-con	HG+GSPE+si-Nrf2
5.34	0.91	3.72	4.13	1.59
6.59	0.98	2.89	3.3	0.99
5.93	1.32	2.26	3.20	1.21
5.32	1.84	4.01	4.65	1.28
6.36	0.99	2.92	3.78	1.00
5.60	1.60	2.53	3.08	1.20
5.29	1.84	4.30	4.64	1.27
6.36	0.98	3.18	3.57	1.00
5.84	1.60	2.23	3.34	1.19

SOD				
control	HG	HG+GSPE	HG+GSPE+si-con	HG+GSPE+si-Nrf2
13.28	6.21	9.67	12.27	5.31
13.03	7.24	11.61	12.82	6.20
13.48	6.86	12.89	13.11	6.31
13.27	6.18	12.21	12.26	6.60
13.03	7.19	12.45	12.83	6.15
13.52	6.84	12.46	13.13	5.63
13.29	6.19	11.92	12.28	7.02
13.03	7.21	10.83	12.81	6.17
13.49	6.85	12.50	13.12	6.29

MDA				
control	HG	HG+GSPE	HG+GSPE+si-con	HG+GSPE+si-Nrf2
3.37	11.45	4.58	5.74	8.78
3.84	8.87	2.92	3.83	7.45
3.98	8.637	4.24	4.10	7.99
3.37	11.33	4.44	6.12	8.76
3.88	9.27	2.59	4.22	7.54
3.61	8.93	3.75	4.08	7.96
3.74	12.08	4.95	4.88	9.45
3.87	10.52	4.00	4.22	7.54
2.88	9.145	4.18	3.31	8.19

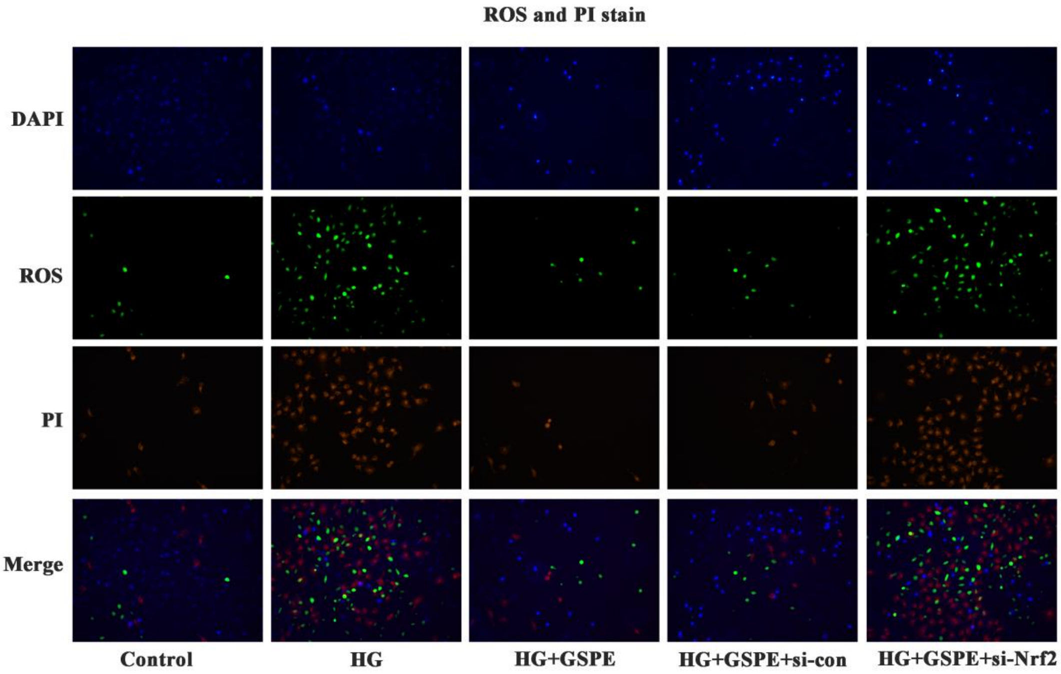
ROS and PI stain
DAPI
ROS
PI
Merge
Control
HG
HG+GSPE
HG+GSPE+si-con
HG+GSPE+si-Nrf2
